# Putative genome features of relic green alga-derived nuclei in dinoflagellates and future perspectives as model organisms

**DOI:** 10.1080/19420889.2020.1776568

**Published:** 2020-06-21

**Authors:** Takuro Nakayama, Kazuya Takahashi, Ryoma Kamikawa, Mitsunori Iwataki, Yuji Inagaki, Goro Tanifuji

**Affiliations:** a Graduate School of Life Sciences, Tohoku University, Sendai, Japan; b Asian Natural Environmental Science Center, The University of Tokyo, Tokyo, Japan; c Graduate School of Agriculture, Kyoto University, Kyoto, Japan; d Center for Computational Sciences, University of Tsukuba, Tsukuba, Japan; e Graduate School of Life and Environmental Sciences, University of Tsukuba, Tsukuba, Japan; f Department of Zoology, National Museum of Nature and Science, Tsukuba, Japan

**Keywords:** Nucleomorph, secondary endosymbiosis, genome reduction, endosymbiotic gene transfer

## Abstract

Nucleomorphs, relic endosymbiont nuclei, have been studied as a model to elucidate the evolutionary process of integrating a eukaryotic endosymbiont into a host cell organelle. Recently, we reported two new dinoflagellates possessing nucleomorphs, and proposed them as new models in this research field based on the following findings: genome integration processes are incomplete, and the origins of the endosymbiont lineages were pinpointed. Here, we focused on the nucleomorph genome features in the two green dinoflagellates and compared them with those of the known nucleomorph genomes of cryptophytes and chlorarachniophytes. All nucleomorph genomes showed similar trends suggesting convergent evolution. However, the number of nucleomorph genes that are unrelated to housekeeping machineries in the two green dinoflagellates are greater than the numbers in cryptophytes and chlorarachniophytes, providing additional evidence that their genome reduction has not progressed much compared with those of cryptophytes and chlorarachniophytes. Finally, potential future work is discussed.

## Main text

Subsequent to primary endosymbiosis between a cyanobacterium and a non-photosynthetic eukaryote, multiple endosymbiotic events between eukaryotes gave rise to various complex plastids on Earth [1]. In the evolutionary processes that integrate two organisms into one, a massive genome reorganization of both the host and endosymbiont-derived genomes are thought to be necessary [2]. The genome reduction of endosymbionts is commonly observed as a result of gene transfer from the endosymbiont to the host genome (i.e., endosymbiotic gene transfers or EGTs) and loss of genes that are unnecessary for the intracellular environment (e.g., mobility and defense mechanisms). In addition, many genes independent from the current plastid origin contribute to the maintenance of plastids (e.g., host-derived and horizontally transferred genes) [2,3]. Through these processes, an endosymbiont was eventually transformed into an organelle with host governance.

Two algal groups, cryptophytes and chlorarachniophytes, have been studied as model organisms to investigate genome integration processes in secondary endosymbiosis due to their retaining relic nuclei derived from eukaryotic endosymbionts (so-called nucleomorphs) [4–6]. On the other hand, a previous study on the nucleomorph genomes of both the lineages indicated that EGTs from the nucleomorphs were likely to have already ceased [4]. Recently, we reported two novel nucleomorph-bearing dinoflagellates for the first time in the last 30 years [7]. These two distantly related dinoflagellates, namely MGD and TGD strains, possess the green alga-derived compartment containing a relic nucleus and a plastid. Transcriptome analyses revealed that some photosynthesis-related proteins of green algal origin are encoded in both nuclear and nucleomorph genomes. This recent finding suggested that genome reorganization via EGTs in both TGD and MGD is still incomplete [7]. Phylogenetic analyses also indicated that the origins of both endosymbionts are close relatives of a particular green algal genus, *Pedinomonas*. Based on these findings, we proposed MGD and TGD as new model organisms to gain insights into the genome evolution associated with eukaryote-eukaryote endosymbiosis. However, the genomic features of MGD and TGD nucleomorphs are yet to be predicted.

Here, we analyzed the features of nucleomorph genomes in MGD and TGD strains. Since the genomic data of MGD/TGD nucleomorphs are currently unavailable, we revisited the transcriptome data reported by Sarai *et al*. (2020) [7]. As described in the previous study, the transcripts of MGD and TGD, which encode proteins with similarity to green algal proteins, could be divided into two populations comprising low and high G + C% [7]. Interestingly, the low G + C% population displayed comparatively high expression levels compared to the other populations. Low G + C% composition is a common feature of previously known nucleomorph genomes and other reduced genomes (e.g., mitochondria, plastids, some parasites and bacterial endosymbionts of insects) [6]. The transcripts belonging to the low G + C% population were predicted to be expressed from the nucleomorph genomes [7]. We classified green algal transcripts into two clusters using an unsupervised clustering method based on the G + C% composition and gene expression levels. In the analysis, 323 and 493 transcripts were predicted as nucleomorph encoding candidates (designated as Nm-candidates in this study) in MGD and TGD, respectively (see Materials and Methods). Among these identified transcripts, 297 and 442 Nm-candidates from the two strains were functionally annotated using the KEGG orthology system (Table 1).

**Table 1.** The putative nucleomorph genome features in green dinoflagellates and the overview of completely sequenced nucleomorph genomes in cryptophytes and chlorarachniophytes.

	MGD*	TGD*	Cryptophytes**	Chlorarachniophytes**
# protein genes	>323	>493	450–500	300–350
# function annotated protein genes	>297	>442	−280	−180
# photosynthesis-related protein genes	>26	>28	0	0
G + C%	36.29***	33.27***	25	30
Gene expression	High	High	High	High
Introns	Probable retained	Probable retained	Retained (excluding an exception****)	Retained

* Number contains only the protein genes with high similarity to green algae.

** Data taken from the review paper (Tanifuji and Onodera 2017).

*** G + C% calculated based on the transcripts of Nm-candidates. Not included intergenic regions.

**** *H. andersenii* (cryptophyte) nucleomorph genome is known as an intron-less eukaryotic genome (Lane et al. 2007).

**Figure 1.**
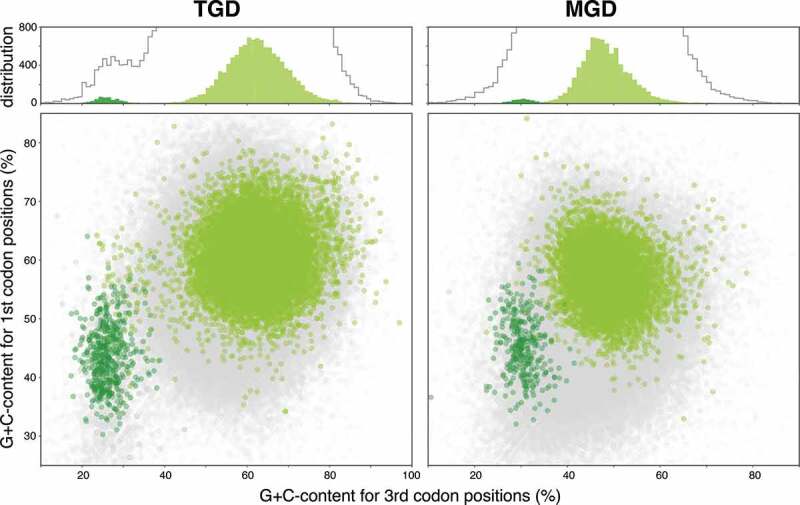
Scatter plots showing results of prediction for nuclear- and nucleomorph-genome coded green algal genes. Light green and dark green markers in the scatter plots indicate G + C% of genes (transcripts) that are predicted to be coded on nuclear-coded genes and Nm-candidates, respectively. Light gray markers on the bottom layer of the scatter plots show G + C% for total protein genes predicted in the transcriptomes. Histograms on upper part show data distribution on G + C% for third codon positions. Light green and dark green bars indicate the distributions of markers with corresponding colors in the scatter plot, while the gray lines in the histograms represent the distribution of the light gray markers.

As of March 2020, four nucleomorph genomes were completely sequenced in each of cryptophytes and chlorarachniophytes [6]. There were about 300–500 non-redundant nucleomorph protein genes set in cryptophytes and chlorarachniophytes [6]. Approximately half of these nucleomorph protein genes are functionally annotated (~280 and 180 protein genes in cryptophytes and chlorarachniophytes, respectively). MGD and TGD contained larger numbers of Nm-candidates with functional annotations compared to those of cryptophytes and chlorarachniophytes (297 and 442 for MGD and TGD, respectively; Table 1). In addition, a large proportion (70–80%; 100–200 genes) of functionally unknown nucleomorph protein genes in cryptophytes and chlorarachniophytes display no similarity to any known protein genes [8,9]. As our prediction focused on genes with probable green algal origins and the genes without sequence similarities to green algal genes in MGD and TGD were undetectable by our strategy, it is reasonable to consider that protein genes without sequence similarity also exist in the MGD and TGD nucleomorph genomes in addition to Nm-candidates detected in this study. G + C% scatter plots for all of the putative protein genes found in TGD and MGD (gray dots in Figure 1) showed that many genes were distributed overlapping the Nm-candidate clusters (dark-green dots in Figure 1). These data suggest that the nucleomorph genome of MGD and TGD contain more nucleomorph protein genes than the Nm-candidate genes and the actual numbers of the nucleomorph genes present in MGD and TGD strains are larger than predicted in our analysis.

Additional insights are provided from the gene content comparison studies. In cryptophytes and chlorarachniophytes, while only a minor proportion of the nucleomorph protein genes (17–31) are associated with plastid function (e.g., genes related to transporter and iron sulfur cluster assembly), no genes are directly related to photosynthesis [6]. Ninety percent of protein genes with functional annotation (180–280) retained in the nucleomorph genomes of cryptophytes and chlorarachniophytes are associated with ‘housekeeping’ functions, such as translation and transcription. Thus, almost all metabolic genes have disappeared from nucleomorph genomes and the majority of current nucleomorph protein genes are retained for a few other essential genes in cryptophytes and chlorarachniophytes. In contrast, 60% of Nm-candidates in MGD and TGD (188 and 263) were assigned to the housekeeping category, and the remaining Nm-candidates were presumably involved in metabolic functions. At least, we observed that 26 and 28 Nm-candidates in MGD and TGD, respectively, play a role relating to thylakoids, essentially involved in photosynthesis (e.g., Photosystem I, II, and Light-harvesting complex proteins). If one assumes that nucleomorph genomes of both MGD and TGD are on the evolutionary trajectories that are similar to those of cryptophytes and chlorarachniophytes, the larger number of metabolic genes present in MGD and TGD nucleomorph genomes might indicate that their genome reduction is still in early stages when compared with those of cryptophytes and chlorarachniophytes.

Another common feature among the nucleomorph genomes of cryptophytes and chlorarachniophytes is the retention of spliceosomal introns despite the extensive genome reductions, with the only exception of *Hemiselmis andersenii* [10]. Similarly, our transcriptome analysis detected 55 MGD and 75 TGD Nm-candidates are involved in splicing-related functions. The abundant Nm-candidates related to splicing suggest that the nucleomorph genomes of MGD and TGD contain spliceosomal introns, as observed in cryptophyte and chlorarachniophyte nucleomorph genomes.

In this study, we predicted the general features of nucleomorph genomes in MGD and TGD. In addition to the low G + C% composition of the genes and relatively high gene expression levels as shown in Sarai *et al*. (2020) [7], genes encoded on the nucleomorph genomes of MGD and TGD are predicted to retain spliceosomal introns, as observed commonly among all known nucleomorph genomes (Table 1). These facts suggest a convergent evolution over the course of eukaryotic genome reduction. On the other hand, the retained larger number of protein genes and their metabolic functions would provide additional evidence for the early state of genome reduction in MGD and TGD as compared to cryptophytes and chlorarachniophytes. Then, what kind of mysteries would be unveiled by the future works using MGD and TGD systems? One of the advantages of MGD and TGD is that the origins of their endosymbionts have been clearly pinpointed to close relatives of the genus *Pedinomonas*, while the plastid origins of cryptophytes and chlorarachniophytes were only narrowed down to the phylum/class-level (Rhodophyta and Chlorophyta/Ulvophyceae, respectively) [7,11]. A comparison between the reduced nucleomorph genomes of the dinoflagellates and that of a closest free-living relative (i.e., *Pedinomonas*) would allow tracing of the detailed processes involved during the reductive genome evolution in eukaryote-eukaryote endosymbioses. A question of interest in nucleomorph biology is why independently reduced genomes show similar trends below. Both cryophytes and chlorarachniophyte nucleomorph genomes show common features, including, 1) elevated AT contents, 2) high gene expression levels (relative to nuclear homologs), 3) retaining spliceosomal introns, 4) genome polyploidy, 5) both the genomes comprise abundant housekeeping genes and a few plastid-associated genes, 6) many protein genes with no similarity to any known proteins, 7) both comprise three linear chromosomes, and 8) existence of sub-telomeric rRNA operons. In this study, we predicted that MGD and TGD nucleomorph genomes are AT-rich (feature 1), have high gene expression levels (feature 2), and retaining spliceosomal introns (feature 3). However, it remains unclear if other features are also common among the four lineages. Future comparative nucleomorph genomics would assist in solving whether those features are also common constraints during endosymbiotic evolution in eukaryotes, and if so, the reason why these features are exhibited.

We identified that genes of certain photosynthetic proteins exist in both the nucleomorph and in the nuclear genomes [7,11]. These genes are considered to be in the midst of undergoing gene transfer process. If a nuclear counterpart of a gene could functionally cover the corresponding nucleomorph homolog, the nucleomorph-counterpart might be lost and then the EGT process for the gene is completed. On the other hand, if the nuclear homolog was not fully established at the end, the homolog would remain nonfunctional in the nuclear genome. Such nonfunctional sequences derived from the nucleomorph genome could disappear or might become a source for invention of novel genes in the nuclear genome. In any event, the photosynthetic genes duplicated between the nucleomorph and the nuclear genomes in MGD and TGD might be an indication of practical examples of ongoing gene transfer from the relic green algal nuclei. In this sense, EGT processes in MGD and TGD can be referred to as “incomplete.” On the other hand, it remains unclear whether abundant EGTs would further occur in the future. Curtis *et al*. (2012) [4] carried out comparative genomics of the representative species of cryptophytes and chlorarachniophytes, and showed that the DNA sequences, which are identical to the nucleomorph genome sequence (i.e., NuNm), are absent from both the nuclear genomes. The short DNA sequences, which are almost identical to the organelle DNA, are referred to as being associated with the frequencies of organelle DNA transfer [12]. Thus, the absence of NuNm in both cryptophytes and chlorarachniophytes nuclear genomes suggests that the reduction via EGTs is, in fact, “frozen” [4]. Hence, the nuclear genomes of MGD and TGD strains need to be sequenced in order to determine how EGT process is active in these systems.

## Materials and methods

### Functional annotation and gene expression level estimation for transcriptomes of MGD and TGD

Transcriptome assemblies and RNA-seq short reads of MGD and TGD were retrieved from the Dryad Digital Repository (DOI: https://doi.org/10.5061/dryad.k6djh9w38) [13] and the NCBI Sequence Read Archive (SRA) database (Accession IDs: DRX181184 and DRX181185), respectively. The functional annotation for the predicted proteins through KEGG [14] orthology ID assignment was done in the same procedure described in Sarai et al. (2020) [7]. TPM (Transcripts Per Million) value for each transcript was calculated as a proxy of gene expression level using bowtie [15] and RSEM [16].

### Detection of the transcripts with potential green algal origin

BLASTP search using each MGD and TGD sequence as a query against a custom protein database were carried out with an e-value cutoff at 1.0E-10. The database contained genome-wide protein sequences from 129 phylogenetically diverse organisms (48 eukaryotic, 68 bacterial, and 13 archaeal species), and the proteins encoded in the plastid genome of *Pedinomonas minor* (GenBank accession no. NC_016733). As candidate of genes with green algal origins, transcripts were retrieved, of which encoded protein sequences had at least one hit to a sequence from Viridiplantae in their top 5 hits of the BLASTP search. Sequences bearing BLASTP hits with *P. minor* plastid proteins were removed as a possible plastid-genome-encoded protein. To avoid the overestimation of gene numbers due to splice variants from an identical locus, all-against-all BLASTN searches were performed for both TGD and MGD transcriptomes and if a bit-score of a blast hit between two sequences was greater than 100, the sequences were considered as potential splice variants. For each bin of potential splice variants expressed from a single gene, a sequence possessing the longest ORF was used in the downstream analyses as a representative of the bin.

### Clustering of the transcripts

Transcripts showing similarity to viridiplant sequences were further classified into two categories by an unsupervised clustering method. Three features for each transcript (G + C content on the first and the third codon positions in an ORF and natural logarithm of TPM) were extracted from MGD- and TGD-transcriptome data. For each dataset comprised those three features, a Gaussian mixture models were fit to a standardized dataset and then the probability of each transcript belonging to each of two categories was calculated. GaussianMixture object of Scikit-learn (version 0.22) [17] was utilized for this procedure with the following options: 2 for number of components; “tied” for covariance parameter. We identified transcripts that were predicted to be included in a cluster with relative low G + C content with over 0.95 probability as Nm-candidates.
